# Effects of External Laryngeal Manipulation on Cervical Spine Motion during Videolaryngoscopic Intubation under Manual In-Line Stabilization: A Randomized Crossover Trial

**DOI:** 10.3390/jcm10132931

**Published:** 2021-06-30

**Authors:** Yoon Jung Kim, Chahnmee Hur, Hyun-Kyu Yoon, Hyung-Chul Lee, Hee-Pyoung Park, Hyongmin Oh

**Affiliations:** Department of Anesthesiology and Pain Medicine, Seoul National University Hospital, Seoul National University College of Medicine, 101, Daehak-ro, Jongno-gu, Seoul 03080, Korea; imovax4@naver.com (Y.J.K.); chahnmee.hur@gmail.com (C.H.); hyunkyu18@gmail.com (H.-K.Y.); lucid80@gmail.com (H.-C.L.); hppark@snu.ac.kr (H.-P.P.)

**Keywords:** external laryngeal manipulation, cervical spine motion, intubation, videolaryngoscopy

## Abstract

We hypothesized that external laryngeal manipulation would reduce cervical spine motion during video laryngoscopic intubation under manual in-line stabilization by reducing the force required to lift the videolaryngoscope. In this randomized crossover trial, 27 neurointerventional patients underwent two consecutive videolaryngoscopic intubation attempts under manual in-line stabilization. External laryngeal manipulation was applied to all patients in either the first or second attempt. In the second attempt, we tried to reproduce the percentage of glottic opening score obtained in the first attempt. Primary outcomes were cervical spine motion during intubation at the occiput-C1, C1–C2, and C2–C5 segments. The intubation success rate (secondary outcome measure) was recorded. Cervical spine motion during intubation at the occiput-C1 segment was significantly smaller with than without external laryngeal manipulation (7.4° ± 4.6° vs. 11.5° ± 4.8°, mean difference −4.1° (98.33% confidence interval −5.8° to −2.3°), *p* < 0.001), showing a reduction of 35.7%. Cervical spine motion during intubation at the other segments was not significantly different with versus without external laryngeal manipulation. All intubations were achieved successfully regardless of the application of external laryngeal manipulation. External laryngeal manipulation is a useful method to reduce upper cervical spine motion during videolaryngoscopic intubation under manual in-line stabilization.

## 1. Introduction

Excessive cervical spine motion during intubation can cause adverse events, such as spinal cord injury, in patients with cervical spine instability [1,2]. Therefore, cervical immobilization with a cervical collar or manual in-line stabilization (MILS) is recommended to reduce cervical spine motion during intubation in patients at risk of cervical spine instability [3,4,5]. In addition, other devices, including videolaryngoscopes, lighted stylets, flexible fiberoptic bronchoscopes, and supraglottic airway devices, are commonly used in such patients instead of direct laryngoscopes to facilitate intubation and reduce cervical spine motion during intubation [6,7,8,9,10,11].

Videolaryngoscopes can provide a better laryngeal view than direct laryngoscopes in patients under cervical immobilization [12,13]. Moreover, various videolaryngoscopes are known to produce smaller cervical spine motion during intubation than direct laryngoscopes [6,8,9,11,14]. However, it is inevitable that some degree of cervical spine motion will occur during videolaryngoscopic intubation even under cervical immobilization [6,8,9,11,14,15,16,17,18]. This is because the force required to lift the videolaryngoscope causes cervical spine motion during intubation, although it is smaller than the force required to lift the direct laryngoscope [19,20,21,22].

External laryngeal manipulation (ELM), which presses the thyroid cartilage backward, is commonly applied to improve the laryngeal view during direct laryngoscopic intubation [23,24,25]. In addition, ELM can increase the percentage of glottic opening (POGO) score during videolaryngoscopic intubation [26]. We expected that ELM, which is preemptively applied before lifting the videolaryngoscope to expose the glottis, would reduce cervical spine motion during videolaryngoscopic intubation by reducing the force required to lift the videolaryngoscope to obtain a laryngeal view which is similar to that obtained during videolaryngoscopic intubation without ELM.

We hypothesized that cervical spine motion during videolaryngoscopic intubation would be smaller with ELM than without ELM in patients under MILS. This study was performed to compare cervical spine motion during videolaryngoscopic intubation under MILS with versus without ELM by analyzing lateral cervical spine radiographs taken before and during intubation.

## 2. Materials and Methods

### 2.1. Ethics

Prior to patient recruitment, this randomized crossover trial was approved by the Institutional Review Board of Seoul National University College of Medicine/Seoul National University Hospital (number H-2004-096-1117, 1 June 2020, 101, Daehak-ro, Jongno-gu, Seoul, Korea). This study was registered at the Clinical Research Information Service, part of the World Health Organization International Clinical Trials Registry Platform (number KCT0005099, 9 June 2020, principal investigator Hyongmin Oh). This study was performed in compliance with Good Clinical Practice guidelines and this paper was written in accordance with the applicable Consolidated Standard of Reporting Trials guidelines. Written informed consent was obtained from all patients prior to participation in this study.

### 2.2. Participants

Patients aged between 20 and 79 years scheduled to undergo elective endovascular cerebral aneurysm coiling under general anesthesia were included in this study. Patients with upper airway lesions (tumor, polyp, trauma, abscess, etc.), cervical spine disease, a history of interventions on the upper airway or cervical spine, coagulopathy, high risk of aspiration (gastrointestinal reflux disease, etc.), or body mass index higher than 30 kg m^−2^ were excluded from the study.

### 2.3. Interventions

All procedures in the study protocol were conducted by one skilled anesthesiologist with experience of more than 50 successful videolaryngoscopic intubations with the aid of two assistants who did not know the study hypothesis, who were the same in both groups except for the application order of ELM. A videolaryngoscope (AceScope™, ACE Medical, Seoul, Korea) with a disposable blade (AceBlade™, ACE Medical, MAC 4 for males and MAC 3 for females) and a reinforced endotracheal tube (Mallinckrodt™, Medtronic, Minneapolis, MN, USA) (internal diameter 7.5 mm for males and 7.0 mm for females), which was mounted on a malleable stylet and angulated 60° at the proximal margin of the cuff, were used. All lateral cervical spine radiographs were taken using a biplanar angiographic unit (Integris Allura™, Philips, Amsterdam, The Netherlands).

Before anesthetic induction, airway-related parameters (Mallampati class, interincisor gap, thyromental distance, sternomental distance, and neck circumference) were measured in the sitting position. After sufficient pre-oxygenation in the supine position, total intravenous anesthesia was induced and maintained by target-controlled infusion of propofol (Fresofol MCT injection, Fresenius Kabi Korea, Seoul, Korea) and remifentanil (Remiva injection, Hana pharm, Seoul, Korea). To facilitate intubation, 0.6 mg kg^−1^ of rocuronium (Esmeron injection, MSD Korea, Seoul, Korea) was administered after loss of consciousness. To alleviate the hemodynamic response to videolaryngoscopic manipulation, 10% lidocaine (Angelcaine Spray, Dong In Dang Pharmaceutical, Siheung, Korea) was sprayed on the upper airway gently exposed using the videolaryngoscope. The patient’s head was placed in the neutral position on a pillow 5 cm in height and MILS was applied by the first assistant to minimize head and neck motion until intubation was actually complete. A lateral cervical spine radiograph was taken before the first attempt.

In the ELM first group, intubation was first attempted with ELM. The videolaryngoscope was lifted only enough to expose the arytenoid cartilage after placing the blade tip at the vallecula. ELM was preemptively applied by the anesthesiologist followed by the second assistant before lifting the videolaryngoscope to expose the glottis, maximizing the POGO score on the videolaryngoscopic monitor. After recording the first POGO score, the endotracheal tube was advanced until its tip reached the vocal cords. A lateral cervical spine radiograph was taken during the first attempt, and the endotracheal tube was gently withdrawn from the upper airway. After bag-valve-mask ventilation for one minute, the patient’s head was placed again in the neutral position on the same pillow. After taking a lateral cervical spine radiograph, intubation was performed in the second attempt without ELM. The videolaryngoscope was lifted only enough to reproduce the first POGO score. Despite best efforts to increase the POGO score with videolaryngoscopic manipulation, if the first POGO score could not be achieved, the second POGO score was noted at that time. After recording the second POGO score, the endotracheal tube was advanced again until its tip reached the vocal cords. A lateral cervical spine radiograph was taken during the second attempt, and the endotracheal tube was gently inserted into the trachea after removing the stylet.

In the ELM second group, the videolaryngoscope was lifted without ELM to maximize the POGO score on the videolaryngoscopic monitor in the first attempt. In the second attempt, the videolaryngoscope was lifted only enough to expose the arytenoid cartilage and ELM was preemptively applied by the anesthesiologist followed by the second assistant before lifting the videolaryngoscope to expose the glottis, reproducing the first POGO score. Despite best efforts to increase the POGO score with ELM, if the first POGO score could not be achieved, the videolaryngoscope was lifted further, only enough to reproduce the first POGO score.

In the intubation attempt with ELM, the anesthesiologist first applied ELM to achieve the desired POGO score and asked the second assistant to switch hands and apply ELM so that the POGO score was maintained. If the POGO score changed during the process of switching hands, feedback from the anesthesiologist was provided to the second assistant, and therefore ELM could be adjusted to achieve the desired POGO score again.

In all patients, intubation was actually performed only in the second attempt, and not in the first attempt. If the intubation time, which was defined as the time interval between insertion of the blade tip into the oral cavity and advancement of the endotracheal tube tip to the vocal cords, exceeded 90 s, or if the peripheral oxygen saturation, which was measured using pulse oximetry, decreased to less than 90%, the attempt was stopped, and rescue bag-valve-mask ventilation was conducted again until the peripheral oxygen saturation reached 100%. After endovascular cerebral aneurysm coiling, all patients were extubated and transferred to the postanesthesia care unit.

### 2.4. Measurement

**Figure 1:**
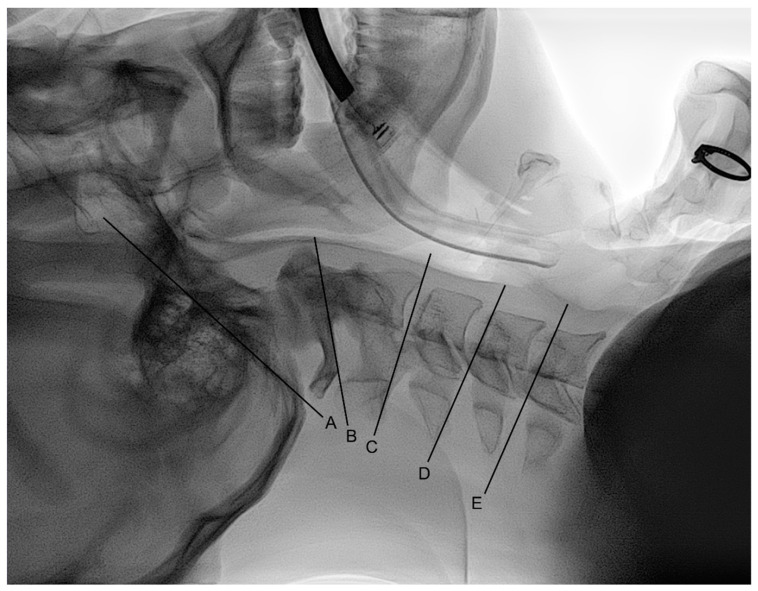
Reference lines of the: (**A**) occiput; (**B**) C1; (**C**) C2; (**D**) C4; (**E**) C5. The reference lines of the occiput and C1 were defined as a line connecting the sellar base and the opisthion and a line connecting the inferior cortical margin of the C1 anterior arch and the inferior cortical margin of the C1 spinous process, respectively [15,16,27]. The reference lines of C2, C4, and C5 were defined as a line connecting the antero-inferior cortical margin of the C2 body and the inferior cortical margin of the C2 spinous process and lines parallel to the endplate of the C4 and C5 body, respectively [15,16,27].

All lateral cervical spine radiographs were automatically saved in the Picture Archiving and Communication System (IFINITT PACS version 5.0.0.143, Infinitt Healthcare, Seoul, Korea) and analyzed by an anesthesiologist who did not know the study hypothesis and the group allocation. The reference lines of the occiput and C1 were defined as a line connecting the sellar base and the opisthion and a line connecting the inferior cortical margin of the C1 anterior arch and the inferior cortical margin of the C1 spinous process, respectively (Figure 1) [15,16,27]. The reference lines of C2, C4, and C5 were defined as a line connecting the antero-inferior cortical margin of the C2 body and the inferior cortical margin of the C2 spinous process and lines parallel to the endplate of the C4 and C5 body, respectively [15,16,27]. Four cervical spine angles before and during intubation were measured between the reference lines of the occiput and C1, C1 and C2, C2 and C5, and C4 and C5.

### 2.5. Outcomes

The primary outcome measure was cervical spine motion during intubation, which was defined as the cervical spine angle during intubation minus the cervical spine angle before intubation, at the occiput-C1, C1–C2, and C2–C5 segments. The secondary outcome measures were the intubation success rate, intubation time, POGO score during intubation, and cervical spine motion during intubation at the C4–C5 segment.

### 2.6. Sample Size

In a previous study, the mean and standard deviation of cervical spine motion during videolaryngoscopic intubation without ELM were 10.4° and 4.4°, respectively, at the occiput–C1 segment in patients under cervical immobilization [15]. Assuming that a 30% reduction in cervical spine motion during intubation at the occiput-C1 segment is clinically significant and there is no significant carryover effect of the application order of ELM on cervical spine motion during intubation, the required sample size for this randomized crossover trial was calculated as 25 patients when setting the α, β, and effect size to 0.017 (0.05/3), 0.2, and 0.7, respectively. Considering a dropout rate of 10%, a total of 28 patients were required for this study.

### 2.7. Trial Design, Randomization, and Blinding

Randomization with computer-generated four-sized blocks was performed by an anesthesiologist who was not involved in the study. Patients were randomly allocated to either the ELM first group (the first intubation attempt with ELM and the second intubation attempt without ELM) or the ELM second group (the first intubation attempt without ELM and the second intubation attempt with ELM) at a ratio of 1:1 based on the allocation sequence made by block randomization. The allocation sequence was sealed in an opaque envelope and released only before anesthetic induction by a nurse who was not involved in the study.

### 2.8. Statistical Methods

Numerical data (proportion) are presented for categorical variables and mean ± standard deviation or median (interquartile range) for continuous variables based on the normality of their distribution. The normality of the data distribution was evaluated by the Shapiro–Wilk test. The Pearson’s χ^2^ test or Fisher’s exact test was used for categorical variables depending on the expected count of the cells. When comparing data according to the application order of ELM, the Student’s *t*-test or Mann–Whitney U test was used for continuous variables depending on the normality of their distribution. When comparing data according to the application of ELM, the paired *t*-test or Wilcoxon signed-rank test was used for continuous variables depending on the normality of their distribution. The effect of ELM and individual patient on cervical spine motion during intubation at the occiput–C1, C1–C2 and C2–C5 segments was analyzed using a linear mixed model. In linear mixed model analysis, the application and application order of ELM and the interaction between them were considered to have fixed effects on cervical spine motion during intubation, whereas individual patient was considered to have a random effect on cervical spine motion during intubation. Basically, a *p*-value less than 0.05 was taken to indicate statistical significance. For cervical spine angle and motion at the occiput–C1, C1–C2, and C2–C5 segments, a *p*-value less than 0.017 (0.05/3) was taken to indicate statistical significance to compensate for multiple comparisons. All statistical analyses were performed using a statistical software (IBM^®^ SPSS^®^ statistics 25, International Business Machines Corporation, Armonk, NY, USA).

## 3. Results

### 3.1. Demographic Characteristics

**Figure 2:**
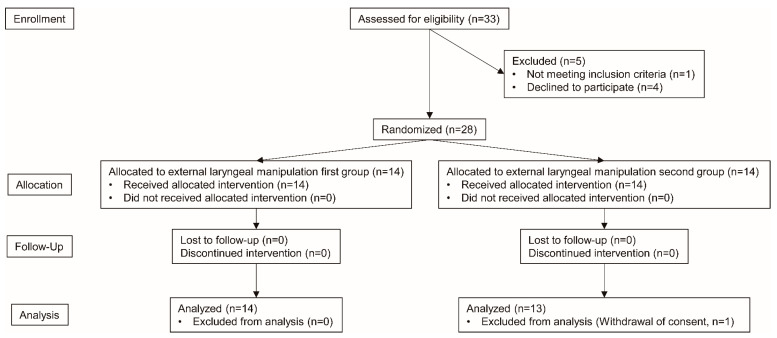
Consolidated Standard of Reporting Trials diagram.

Among a total of 33 patients who were assessed for eligibility between July 2020 and October 2020, five patients were excluded, and the remaining 28 patients were randomized into the two groups (Figure 2). After randomization, one patient in the ELM second group was excluded from data analysis because consent to participate in the study was withdrawn. There were no significant differences in demographic or airway-related data between the ELM first and ELM second groups (Table 1).

### 3.2. Cervical Spine Motion

In linear mixed model analysis, the application of ELM was significantly associated with cervical spine motion during intubation at the occiput-C1 (*p* < 0.001) segment, but not at the C1–C2 (*p* = 0.255) and C2–C5 (*p* = 0.045) segments. There was no significant carryover effect of the application order of ELM on cervical spine motion during intubation at the occiput–C1 (*p* = 0.818), C1–C2 (*p* = 0.134), and C2–C5 (*p* = 0.793) segments. A significant random effect of individual patient on cervical spine motion during intubation was observed at the occiput–C1 (*p* = 0.006) segment, but not at the C1–C2 (*p* = 0.054) and C2–C5 (*p* = 0.175) segments.

**Figure 3:**
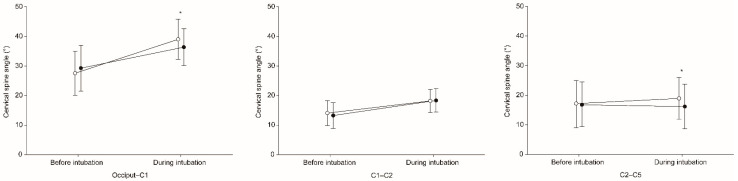
Cervical spine angle before and during intubation at the occiput-C1, C1–C2, and C2–C5 segments. * Means a significant difference in cervical spine angle with ELM (black colored circle) versus without ELM (white colored circle) external laryngeal manipulation.

Significantly smaller cervical spine motion during intubation occurred with versus without ELM at the occiput–C1 segment (7.4° ± 4.6° vs. 11.5° ± 4.8°, mean difference −4.1° (98.33% confidence interval −5.8° to −2.3°), *p* < 0.001), representing a reduction of 35.7% (Table 2). Cervical spine motion during intubation was not significantly different with versus without ELM at the C1–C2, C2–C5, and C4–C5 segments. Cervical spine angle before intubation did not significantly differ with versus without ELM (Figure 3). Cervical spine angle during intubation was significantly smaller with ELM than without ELM at the occiput–C1 (36.5° ± 6.3° vs. 39.1° ± 6.8°, mean difference −2.6° (98.33% confidence interval −4.4° to −0.8°), *p* = 0.001) and C2–C5 (16.0° ± 7.6° vs. 18.7° ± 7.2°, mean difference −2.7° (98.33% confidence interval −4.9° to −0.5°), *p* = 0.004) segments, but not at the C1–C2 and C4–C5 segments.

### 3.3. Intubation Performance

All intubation was achieved successfully regardless of the application of ELM, but the intubation time was significantly longer with ELM than without ELM (33.0 (25.0 to 43.0) vs. 26.0 (20.0 to 35.0) s, mean difference 6.5 (95% confidence interval 3.0 to 11.0) s, *p* = 0.002) (Table 3). The POGO score obtained in the first attempt without ELM was reproduced in the second attempt with ELM in all patients in the ELM second group. In contrast, in seven patients in the ELM first group, the POGO score obtained in the first attempt with ELM was not reproduced in the second attempt without ELM, resulting in a significantly higher POGO score with ELM (54.3% ± 25.1% vs. 27.9% ± 26.4%, mean difference 26.4% (95% confidence interval 3.4% to 49.5%), *p* = 0.031). This made a significantly higher POGO score with ELM in total patients (59.3% ± 28.0% vs. 52.4% ± 31.8%, mean difference 6.9% (95% confidence interval 0.2% to 13.5%), *p* = 0.044).

## 4. Discussion

A reduction in cervical spine motion during intubation is particularly important in patients at risk of cervical spine instability. In this study, we compared cervical spine motion during videolaryngoscopic intubation under MILS at four cervical spine segments with versus without ELM. When ELM was applied during videolaryngoscopic intubation under MILS, cervical spine motion was significantly reduced by 36% at the occiput-C1 segment.

ELM is usually applied when the glottis is not exposed despite maximal lifting of the laryngoscope during intubation [23,24,25,26]. In this study, ELM was preemptively applied to reduce cervical spine motion during intubation by reducing the force required to lift the videolaryngoscope. This study showed that, even with efforts to minimize cervical spine motion during intubation such as applying MILS and using a videolaryngoscope, ELM additionally reduced cervical spine motion during intubation at the occiput-C1 segment by 4.1°. However, ELM presses the thyroid cartilage backward at the level of C4 or C5, which can induce cervical spine motion by exerting a direct force on the cervical spine in patients with lower cervical spine instability. Although there was no significant difference in cervical spine motion during intubation at the C2–C5 segment, including the C4–C5 segment, with versus without ELM in this study, it must be taken into account that this study was conducted in patients without cervical spine instability. Therefore, care is required in its application in patients at risk of lower cervical spine instability.

In patients at risk of cervical spine instability, MILS has commonly been recommended and applied to reduce cervical spine motion during intubation [3,4]. In a previous study, MILS was reported to reduce head extension during direct laryngoscopic intubation by 4–5° [28]. In another previous study, MILS reduced upper cervical spine motion during videolaryngoscopic intubation by 4° as compared with cervical immobilization with a cervical collar [29]. However, in other previous studies, MILS worsened the laryngeal view and increased the intubation failure rate, intubation time, and force applied to airway tissues by the blade during direct laryngoscopic intubation [30,31]. Thus, MILS may be more suitable for intubation using other devices that do not require direct exposure of the glottis, such as videolaryngoscopes, rather than direct laryngoscopic intubation [32].

Videolaryngoscopic intubation has advantages with regard to the force required to lift the laryngoscope and cervical spine motion during intubation as compared with direct laryngoscopic intubation. In previous studies comparing the lifting force during intubation between a direct laryngoscope and a videolaryngoscope, the peak and average lifting forces were significantly reduced by about half when using a videolaryngoscope in patients expected to have either a normal or difficult airway [20,21,22]. With the same context, numerous previous studies comparing cervical spine motion during intubation between direct laryngoscopes and various videolaryngoscopes, have reported significantly smaller cervical spine motion during videolaryngoscopic intubation [6,8,9,11,14,17,18]. Nevertheless, intubation using various videolaryngoscopes produced cervical spine motion of 3–13° at the occiput-C1 segment even under MILS in previous studies [8,9,11,17]. Previous studies ave shown that the use of other devices, such as fiberoptic bronchoscopes, optic stylets, and lighted stylets, could reduce cervical spine motion during intubation as compared with the use of laryngoscopes, but the use of such devices also could not avoid some degree of cervical spine motion during intubation [10,11,15,33].

In this study, in the ELM first group, the first POGO score was not reproduced in the second attempt in seven patients, leading to a significantly higher POGO score with than without ELM. This finding supports the results of previous studies that ELM improved the laryngeal view during videolaryngoscopic intubation [26]. However, on the one hand, the median intubation time was 7 s longer with ELM than without ELM, which is considered to have been because the process of applying ELM optimally was added to the process of videolaryngoscopic intubation. In addition, it may indicate that a 7% higher mean POGO score with ELM was insufficient to improve the difficulty of videolaryngoscopic intubation or shorten the intubation time. On the other hand, in patients whose first POGO score was not reproduced in the second attempt, the intubation time was comparable with versus without ELM, which is thought to have been due to the relatively long time taken for videolaryngoscopic manipulation.

This study had several limitations. First, the anesthesiologist was not blinded to the application of ELM, which could have led to a potential bias. Second, in this study, intubation was performed only by one skilled anesthesiologist using AceScope™ with AceBlade™, a videolaryngoscope with a Macintosh type blade. Therefore, our results may not be reproduced when intubation is performed by anesthesiologists less familiar with videolaryngoscopic intubation or using other videolaryngoscopes with angulated or channeled blades. Further studies are necessary to determine whether ELM can reduce cervical spine motion during intubation by novice anesthesiologists or using such videolaryngoscopes. Third, in this study, all lateral cervical spine radiographs were taken intermittently to avoid excessive radiation exposure. More detailed information about cervical spine motion during intubation could have been obtained if all lateral cervical spine radiographs had been taken continuously. Fourth, the random effect of individual patient on cervical spine motion during intubation at the occiput-C1 segment was significant in linear mixed model analysis. Thus, our results may not be reproduced in other patients due to individual variations in the structure and function of the cervical spine. Moreover, since this study was conducted in patients without cervical spine instability, it is difficult to guarantee that similar results would be shown in patient with cervical spine instability. Further studies are required to determine the generalizability and expandability of our results. Fifth, because the sample size may not have been large enough to properly test the carryover effect of the application order of ELM on cervical spine motion during intubation or to sufficiently investigate the effect of ELM on the intubation success rate, our results associated with these should be interpreted with caution. Sixth, we assumed that smaller cervical spine motion would reduce the risk of adverse events during intubation, but it is not known how many degrees of cervical spine motion is actually dangerous. Lastly, we did not directly measure the force required to press the thyroid cartilage during ELM, the force required to lift the videolaryngoscope during intubation, and the force required to apply MILS.

## 5. Conclusions

The results of the present study showed that videolaryngoscopic intubation under MILS led to significantly smaller cervical spine motion at the occiput-C1 segment with ELM than without ELM. This suggested that ELM would be a useful method to reduce upper cervical spine motion when videolaryngoscopic intubation is performed under MILS.

## Figures and Tables

**Table 1:** Patient characteristics.

	ELM First (*n* = 14)	ELM Second (*n* = 13)
Demographic data		
Male sex	6 (42.9%)	4 (30.8%)
Age (year)	56.7 ± 8.7	60.3 ± 12.3
Height (cm)	163.8 ± 10.5	160.6 ± 6.4
Weight (kg)	67.4 ± 17.6	63.8 ± 7.1
Body mass index (kg m^−2^)	24.2 (21.4 to 26.0)	23.4 (22.3 to 27.3)
ASA physical status		
I	4 (28.6%)	2 (15.4%)
II	5 (35.7%)	10 (76.9%)
III	5 (35.7%)	1 (7.7%)
Airway-related data		
Mallampati class		
I	3 (21.4%)	4 (30.8%)
II	5 (35.7%)	3 (23.1%)
III	5 (35.7%)	6 (46.2%)
IV	1 (7.1%)	0 (0.0%)
Interincisor gap (cm)	4.8 (4.0 to 5.0)	5.0 (4.0 to 5.3)
Thyromental distance (cm)	8.5 (8.0 to 9.0)	9.0 (8.0 to 9.0)
Sternomental distance (cm)	16.0 (15.0 to 16.5)	16.0 (15.5 to 18.5)
Neck circumference (cm)	36.5 (33.5 to 40.5)	35.0 (34.0 to 36.0)

Data were presented as number (proportion), mean ± standard deviation, or median (interquartile range). ELM, external laryngeal manipulation; ASA, American Society of Anesthesiologists.

**Table 2:** Cervical spine motion during intubation.

	With ELM	Without ELM	Mean Difference (98.33% CI)	*p*-Value
Total patients (*n* = 27)				
Occiput–C1 (°)	7.4 ± 4.6	11.5 ± 4.8	−4.1 (−5.8 to −2.3)	<0.001
C1–C2 (°)	5.7 ± 4.1	4.7 ± 3.5	1.0 (−1.1 to 3.0)	0.244
C2–C5 (°)	−0.9 ± 4.4	1.3 ± 4.4	−2.2 (−4.8 to 0.4)	0.040
C4–C5 (°)	−0.4 ± 1.9	−0.8 ± 2.2	0.4 (−0.7 to 1.6)	0.343
Patients whose first POGO score was reproduced in second attempt (*n* = 20)				
Occiput–C1 (°)	8.0 ± 4.9	11.8 ± 4.5	−3.8 (−6.0 to −1.6)	<0.001
C1–C2 (°)	6.1 ± 4.3	4.9 ± 3.5	1.2 (−1.3 to 3.7)	0.229
C2–C5 (°)	−1.8 ± 4.5	0.7 ± 4.7	−2.5 (−5.9 to 0.8)	0.064
C4–C5 (°)	−0.5 ± 1.9	−1.0 ± 2.2	0.5 (−0.8 to 1.7)	0.356
Patients whose first POGO score was not reproduced in second attempt (*n* = 7)				
Occiput–C1 (°)	5.7 ± 3.2	10.6 ± 6.0	−4.9 (−9.1 to −0.7)	0.008
C1–C2 (°)	4.4 ± 3.2	4.1 ± 3.4	0.3 (−4.9 to 5.5)	0.850
C2–C5 (°)	1.6 ± 3.1	2.9 ± 2.8	−1.3 (−6.3 to 3.7)	0.433
C4–C5 (°)	0.1 ± 2.1	−0.4 ± 2.2	0.4 (−3.6 to 4.4)	0.740

Data were presented as mean ± standard deviation. *p*-values less than 0.0167 (0.05/3) were considered statistically significant to compensate for multiple comparisons of primary outcome. ELM, external laryngeal manipulation; CI, confidence interval; POGO, percentage of glottic opening.

**Table 3:** Intubation performance.

	With ELM	Without ELM	Effect Size * (95% CI)	*p*-Value
Total patients (*n* = 27)				
Intubation success	27 (100%)	27 (100%)	Not applicable	1.000
Intubation time (s)	33.0 (25.0 to 43.0)	26.0 (20.0 to 35.0)	6.5 (3.0 to 11.0)	0.002
POGO score (%)	59.3 ± 28.0	52.4 ± 31.8	6.9 (0.2 to 13.5)	0.044
Patients whose first POGO score was reproduced in second attempt (*n* = 20)				
Intubation success	20 (100%)	20 (100%)	Not applicable	1.000
Intubation time (s)	32.0 (24.3 to 42.5)	26.0 (18.5 to 33.0)	7.0 (3.5 to 11.5)	0.001
POGO score (%)	61.0 ± 29.4	61.0 ± 29.4	Not applicable	1.000
Patients whose first POGO score was not reproduced in second attempt (*n* = 7)				
Intubation success	7 (100%)	7 (100%)	Not applicable	1.000
Intubation time (s)	37.0 (28.0 to 56.0)	26.0 (24.0 to 43.0)	4.5 (−11.0 to 20.5)	0.499
POGO score (%)	54.3 ± 25.1	27.9 ± 26.4	26.4 (3.4 to 49.5)	0.031

Data were presented as number (proportion), mean ± standard deviation, or median (interquartile range). * Indicates mean difference or median difference. ELM, external laryngeal manipulation; CI, confidence interval; POGO, percentage of glottic opening.

## Data Availability

The data presented in this study are available on request from the corresponding author. The data are not publicly available due to privacy and ethical restrictions.
